# Identification and characterisation of diabetes in Uganda: protocol for the nested, population-based ‘Diabetes in low-resource Populations’ (DOP) Study

**DOI:** 10.1136/bmjopen-2023-071747

**Published:** 2023-09-13

**Authors:** Isaac Sekitoleko, Wisdom P Nakanga, Emily Webb, Viola Mugamba, Priscilla Balungi, Bernard Mpairwe, Ongaria Terry, Ronald Makanga, Esther Nabanoba, Joseph O Mugisha, Geofrey Kimbugwe, Moffat J Nyirenda, Anxious J Niwaha

**Affiliations:** 1 Statistics and Data Science, Medical Research Council/Uganda Virus Research Institute and LSHTM Uganda Research Unit, Entebbe, Uganda; 2 Non-communicable diseases, Malawi Epidemiology and Intervention Research Unit (MEIRU), Chilumba, Malawi; 3 Faculty of Infectious and Tropical Diseases, London School of Hygiene and Tropical Medicine, London, UK; 4 Non-communicable diseases Theme, Medical Research Council/Uganda Virus Research Institute and LSHTM Uganda Research Unit, Entebbe, Uganda; 5 NCD Epidemiology, London School of Hygiene and Tropical Medicine, London, UK

**Keywords:** DIABETES & ENDOCRINOLOGY, EPIDEMIOLOGY, General diabetes

## Abstract

**Introduction:**

Sub-Saharan Africa is experiencing an increasing burden of diabetes, but there are little reliable data, particularly at the community level, on the true prevalence or why this condition affects young and relatively lean individuals. Moreover, the detection of diabetes in Africa remains poor, not only due to a lack of resources but because the performance of available diagnostic tests is unclear.

**Methods:**

This research aims to (1) determine the prevalence and risk factors of diabetes in a rural Ugandan population, (2) use clinical and biochemical markers to define different diabetes phenotypes and (3) study the progression of diabetes in this population. We will also assess the utility of the widely used tests (glycated haemoglobin (HbA1c), oral glucose tolerance test (OGTT) and fasting glucose) in diagnosing diabetes.

**Design:**

This is a population-based study nested within the longstanding general population cohort in southwestern Uganda. We will undertake a population survey to identify individuals with diabetes based on fasting glucose, HbA1c, OGTT results or history of pre-existing diabetes.

**Participants:**

The study intends to enrol up to 11 700 individuals aged 18 years and above, residing within the study area and not pregnant or within 6 months post-delivery date. All participants will have detailed biophysical and biochemical/metabolic measurements. Individuals identified to have diabetes and a random selection of controls will have repeat tests to test reproducibility before referral and enrolment into a diabetic clinic. Participants will then be followed up for 1 year to assess the course of the disease, including response to therapy and diabetes-related complications.

**Conclusions:**

These data will improve our understanding of the burden of diabetes in Uganda, the risk factors that drive it and underlying pathophysiological mechanisms, as well as better ways to detect this condition. This will inform new approaches to improve the prevention and management of diabetes.

**Ethics and dissemination:**

This study protocol was approved by the Uganda Virus Research Institute Research Ethics Committee (REC) (number: G.C./127/21/09/858), the London School of Hygiene and Tropical Medicine REC (number: 26638) and the Uganda National Council for Science and Technology (protocol number: HS1791ES). Written informed consent will be obtained from all participants before being enrolled on to the study and conducting study-related procedures. Research findings will be disseminated in policy briefs, seminars, local and international conferences and publications in peer-reviewed open-access journals. As part of the dissemination plans, findings will also be disseminated to patient care groups and to clinicians.

**Trial registration number:**

NCT05487079.

**STRENGTHS AND LIMITATIONS OF THIS STUDY**

- The large sample size gives sufficient power to answer the primary and secondary research questions.
- This project will provide a characterised cohort of individuals with diabetes who will be followed up for future research.
- Biospecimens will be stored for future research purposes.
- This study is nested in a longstanding and well-characterised general population cohort with routine follow-ups.
- Participants could be lost to follow-up which may impact on the study power and may introduce bias.

## Introduction

Diabetes is a global problem disproportionately affecting low-income and middle-income countries (LMICs). According to the 2021 International Diabetes Federation estimates, 80% of the approximately 537 million adults (20–79 years) living with diabetes reside in LMICs.^1^ In sub-Saharan Africa (SSA), an estimated 24 million people are living with diabetes, which is projected to reach 55 million within 20 years.^1^ Moreover, the region is said to harbour the highest proportion of undiagnosed patients with diabetes.^1^ This poses a major threat to sustained development in the region if resources have to be found to treat the complications of this condition.

An effective response will require a clear understanding of the burden, important local determinants of risk, and pathophysiological and clinical manifestations of diabetes. However, there are limited Africa data, with most coming from relatively small studies using unstandardised methodologies. This may explain the significant variation in diabetes prevalence observed between countries.^2^ Moreover, there are concerns that some of the widely used tests to diagnose and monitor diabetes may be unreliable in Africa. Although still a relatively unexplored area, the clinical utility of glycated haemoglobin (HbA1c) in SSA has been questioned due to the high prevalence of haemoglobinopathies such as sickle cell trait and other medical conditions that might affect test reliability, including anaemia, malaria and HIV infections.^3–6^ Similarly, there are concerns about the utility of oral glucose tolerance test (OGTT), the current gold-standard test for diagnosing diabetes.^7^ It is not only complex and expensive but may have other important limitations, such as the need to have adequate caloric intake in the days preceding the test. Our preliminary study showed that a missed or small meal the night before a test could lead to falsely elevated glucose.^8^ This might explain why some Africa studies have found a high prevalence of diabetes or glucose intolerance where OGTT has been used. Therefore, OGTTs cannot be fully relied on to screen for diabetes in settings where food insecurity or missed evening meals are common.

There is also increasing evidence that diabetes may manifest differently in Africa compared with its presentation in high-income countries. For example, while traditionally, type 2 diabetes is considered a disease of old age associated with obesity and insulin resistance, this condition appears to occur in relatively young and lean individuals in Africa and other LMICs. This evidence initially came from Southeast Asia, where people were observed to develop type 2 diabetes at lower levels of obesity than white Caucasians; this has led to Asia-specific lower body mass index (BMI) thresholds for defining obesity.^9^ The explanation has been that Southeast Asians have a propensity towards central obesity (and therefore insulin resistance) at relatively low body weight. Our group recently showed that 50% and nearly 40% of newly diagnosed type 2 diabetes (negative for pancreatic autoantibodies) in Uganda are younger than 50 years and have a BMI of ≤25 kg/m^2^, respectively.^10^ In contrast to observations in Southeast Asia, the ‘thin’ patients showed no evidence of excess ectopic or visceral fat (using anthropometric and electric bioimpedance measurements) or insulin resistance. Instead, they had features consistent with insulin deficiency (lower fasting insulin, C peptide insulinogenic index and Homeostatic Model Assessment for Insulin Resistance (HOMA-IR)).^11^

These observations raise important research questions and have huge potential clinical implications: what are the mechanisms that drive diabetes in Africa; what are the right approaches to its prevention and management? For example, are the lifestyle interventions targeting weight loss or metformin use (as an insulin sensitiser), two traditionally recommended first-line management options for type 2 diabetes, effective in Africa?

This necessitates rigorous studies to unravel the complex manifestations of diabetes in Africa and identify the right approaches to prevention and management. Therefore, in the Diabetes in low-resource Populations Study, we aim to: (1) determine the prevalence of diabetes in Uganda, (2) identify and characterise the different diabetes phenotypes, and (3) develop a cohort in which we can examine and understand the course or progression of this disorder and its different diabetes phenotypes. All patients except those with a known diagnosis of diabetes (on oral or insulin treatment for diabetes) will undergo OGTT. With this approach, we will be able to compare the diagnostic accuracy of fasting glucose and HbA1c with OGTT.

## Research design and methods

### Study design

This will be a population-based study nested within the general population cohort (GPC)—a longstanding population cohort study in southwestern Uganda.^12^

### Study setting

The study will be conducted in Uganda. Uganda is undergoing urbanisation, but more than 70% of the population still lives in rural areas. The study shall recruit from two sites (rural and periurban areas) across the greater Masaka region in southwest Uganda to explore the differences in lifestyle and how these influence the prevalence of diabetes and its outcomes. The GPC in Kyamulibwa subcounty, Kalungu district in southwest Uganda, will provide the rural population and the town of Lukaya, the periurban population. Both rural and periurban sites are established research areas.

### Sample population

The GPC is a rural population-based open cohort study of people living within the 25 villages of the Kyamulibwa subcounty. The population in Kyamulibwa is assessed routinely during annual surveys as part of the census and 2-year medical surveys. The entire adult population of the GPC will be invited to participate in this study. Data from the most recent census in the GPC study villages show that there are 8864 adults out of the total population of 20 751. A recent enumeration for a COVID-19 survey conducted in Lukaya established that there are approximately 10 000 adults in all the five villages. All these will be invited to take part in our study. The combined adult population within the GPC and Lukaya will give us enough power to rigorously examine our primary objectives, allow explorations of the outcomes and key risk factors, and establish long-term cohorts.

### Study objectives

#### Primary study objective

To determine the prevalence of diabetes and pre-diabetes in a population-based cohort.

#### Secondary study objectives

1. To establish a cohort of well-characterised patients with diabetes to understand disease progression and the course of the different phenotypes, including response to treatment.
2. To identify and recruit a cohort of individuals with pre-diabetes and volunteers without diabetes for longitudinal follow-up.
3. To assess the burden and rates of progression of vascular complications associated with the different phenotypes of diabetes.

#### Primary study outcome

1. Prevalence of diabetes and pre-diabetes.

#### Secondary study outcomes

1. Number of distinct phenotypes of type 2 diabetes within this population.
2. Baseline prevalence of vascular complications among individuals with diabetes.
3. Rate of progression of vascular disease within this population.

### Sampling strategies

**Figure 1:**
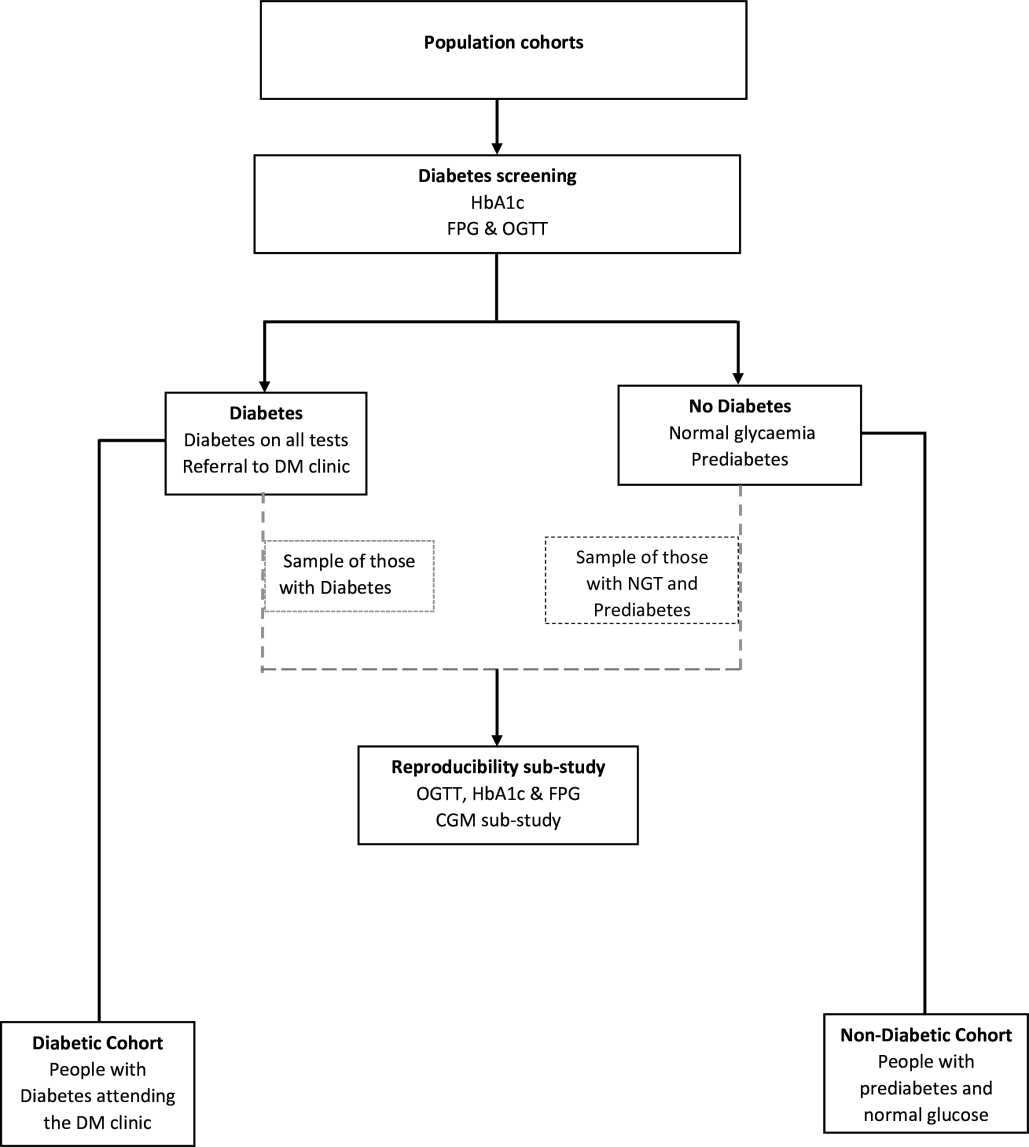
Participant’s flow. CGM, continuous glucose monitoring; DM, diabetes mellitus; FPG, fasting plasma glucose; HbA1c, glycated haemoglobin; NGT, normal glucose tolerance; OGTT, oral glucose tolerance test.

Recruitment will be restricted to individuals who reside within defined study areas. Research team members (mobilisers) will visit each household in the study areas. All adults aged 18 years and above will be invited to take part in the study (table 1). We will recruit every adult who is able and willing to consent and is a resident at a household (ie, not a temporary member with intent to leave) in the study areas. The participant’s flow in the study is shown in figure 1.

**Table 1:** Eligibility criteria for the participants enrolled into the DOP Study

Inclusion criteria	Individuals aged 18 years and above
	Individuals residing within the study area for the past 6 months
	Individuals willing to provide informed consent
Exclusion criteria	Pregnant women and women within 6 months post-delivery
	Participants unwilling to give informed consent
	Living outside the geographical sampling frame for the relevant site

DOP, Diabetes in low-resource Populations.

Study phases: this study will consist of overlapping phases, as described in table 2.

**Table 2:** Implementation phases (I–IV)

Phase I	The first phase involves conducting the main baseline survey, whose outcomes are the prevalence of diabetes, pre-diabetes and associated risk factors, and glucose tolerance subgroups based on WHO criteria. This phase will include the following activities: Research team selection and training Stakeholder and community sensitisation Mobilisation and participant notification Recruitment of participants Obtaining consent and baseline questionnaire data collection Anthropometric measurements Baseline OGTT Blood and urine sample collection We will use systems already in place for the GPC cohort to achieve and maintain high levels of participation. These systems include: GPC census/survey teams, meeting the community advisory board, meeting local leaders, organising community meetings and participant notification.
Phase II	This phase involves the assessment of the reproducibility of the diagnostic approaches in a randomly selected set of participants. In this phase, we shall assess the diagnostic reproducibility of the fasting glucose and OGTT (diagnostic reproducibility substudy). We will determine whether participants with isolated impaired fasting glucose and isolated impaired glucose tolerance have elevated blood glucose in day-to-day living, compared with normoglycaemic individuals using continuous glucose monitoring. This phase will involve the following activities: Repeat OGTT Continuous glucose monitoring
Phase III	This phase aims to assess and describe any diabetes clusters or phenotypes by comparing baseline characteristics, and evaluating the distribution of various measures inclusive of biochemical parameters (eg, fasting and stimulated C peptide), adiposity and anthropometric indices. Further tests, including vascular complications assessment, endogenous insulin secretion measurement and islet autoantibodies profiles, will be done in future to better understand the differences between the various diabetes phenotypes.
Phase IV	In this phase, we aim to establish well-characterised people with and without diabetes. The newly diagnosed participants with diabetes will be referred to the local diabetes clinics, where they will be managed according to the Uganda Ministry of Health guidelines. The newly diagnosed patients with diabetes and those with known diabetes will be followed up in clinics (supported by the research team) to study the *course of the condition*, including; (1) response to treatment: participants will be followed up at the clinic to assess glycaemic control (HbA1c) every 3 months, determine the time to treatment intensification (need for dosage increment, the addition of another drug or switching from oral hypoglycaemic agents to insulin), and (2) assess disease progression *through* (a) measuring of endogenous insulin secretion (using mixed meal tolerance tests), (b) assessment of insulin resistance and repeat vascular complications reassessment (including retinopathy, nephropathy). Concurrently, participants with pre-diabetes and those with normal glucose levels will be followed in a longitudinal study nested in the GPC to determine the incidence of diabetes.

GPC, general population cohort; HbA1c, glycated haemoglobin; OGTT, oral glucose tolerance test.

### Data collection

Data will be collected on social demographics (age, sex and marital status), socioeconomic (household assets, household income, level of education and occupation), lifestyle (diet, physical activity, smoking and alcohol consumption) and family history of the disease (diabetes, hypertension, dyslipidaemia, coronary heart disease, stroke, renal disease, diabetes-related eye disease, HIV status, tuberculosis, malaria and other chronic infections such as hepatitis C virus). Data on biophysical measurements will be collected, including: height, weight, total body fat, skeletal muscle mass, visceral fat, waist and hip circumferences, mid-upper arm circumference (MUAC) and calf circumference, blood pressure and pulse.

### Patient and public involvement

Patients, community representatives and policymakers are engaged at different stages of our research including formulating research questions. We have community advisory groups to facilitate our engagement with the community.

### Study procedures

#### Biophysical measurements

Standing height will be measured with a Seca stadiometer to the nearest 0.1 cm. The sitting height will be determined by measuring the distance from the sitting surface to the top of the head using a Seca stadiometer and a flat-seated seat, with the sitting surface centred and placed against the backboard of the stadiometer. Weight will be measured in light clothing with a Seca 761 mechanical flat scale (spring-type scale) to the nearest 0.1 kg. The scale will be placed level on a hard surface.

Total body fat, skeletal muscle mass and visceral fat will be measured with an OMRON KaradaScan, Body Composition Monitor Bio-impedance scale (OMRON Healthcare Company Limited, Japan). Waist and hip circumferences will be measured with a Seca 201 measuring tape to the nearest 0.1 cm. Waist circumference will be taken from the narrowest part of the abdomen between the ribs and the iliac crest (top of the hip bone). Hip circumference will be measured as the tape is placed around the most protruding part of the buttocks. MUAC will be measured with a MUAC measuring tape to the nearest 0.1 cm at the midpoint level between the tip of the acromion and the olecranon.

Calf circumference will be measured with a Seca 201 measuring tape to the nearest 0.1 cm. The circumference of the calf will be measured at its widest point perpendicular to the long axis of the calf to the nearest 0.1 cm. Skinfold thickness over the triceps muscle will be measured with a Harpenden skinfold calliper. Skinfold thickness will be measured at the posterior surface of the upper arm over the triceps muscle at the midpoint between the acromion and the olecranon process of the right arm. Blood pressure and pulse will be measured three times with resting intervals of 5 min using an automated upper arm blood pressure monitor (OMRON, Healthcare Europe, the Netherlands).

#### Continuous glucose monitoring

Continuous glucose monitoring (CGM) will be carried out using the blinded Freestyle Libre Pro Flash Glucose Monitoring System (Abbott Laboratories, Illinois, USA), a professional CGM device which records interstitial glucose every 15 min for up to 2 weeks.

#### OGTT and biological sample collection

The OGTT will be performed following the WHO guidelines. The study nurse will confirm if the participant fasted overnight for at least 8 hours. Baseline fasting blood samples will be collected for fasting plasma glucose measurement and biobanking for future diabetes and related studies. Following the collection of the fasting blood samples, a standard 75 g of glucose will be administered, and a repeat blood sample will be collected after 120 min. Participants who have not fasted appropriately will be asked to return in a fasted state on another day.

#### Sample processing, storage and transport of samples

Blood samples for glucose measurement will be collected in vacutainers with sodium fluoride, centrifuged and separated into two cryovials (aliquots) immediately and transported in an icebox maintained at 4­8°C to the central laboratory for immediate testing (within 8 hours of collection). Whole blood samples for full blood count and HbA1c will be collected in vacutainers containing EDTA. Plasma glucose, full blood count and HbA1c measurements will be performed at the central biochemistry and clinical diagnostic laboratory services laboratory at the Medical Research Council/Uganda Virus Research Institute (MRC/UVRI) and London School of Hygiene and Tropical Medicine (LSHTM) Uganda Research Unit Entebbe, Uganda. Laboratory analyses will be performed on the Roche Cobas 6000 analyser (Hitachi high technologies corporation, Tokyo, Japan). Plasma glucose will be measured by the glucokinase method. HbA1c will be measured on Cobas 6000 by the immunoassay technique, calibrated to the International Federation of Clinical Chemistry. Serum and plasma samples will be centrifuged immediately and divided into cryovials for long-term storage at −80°C (see table 3 for further details).

**Table 3:** Sample processing and storage

Sample	Processing
5 mL serum separating tube	The tube will be immediately spun, and the serum will be aliquoted into five 2 mL microvials (cryovials). The cryovials will then be placed in a cool box at 4–8°C until shipment to the central laboratory, where they will be stored in a −80°C freezer.
2 mL NaF blood tube (all)	Blood tubes will gently be inverted to mix and then spun within 15 min of blood draw, aliquoted into 2 mL cryovials and then placed in a cool box at 4–8°C until shipment to the central biorepository for measurement of plasma glucose.
2 mL EDTA	The tube will be inverted gently to mix. This tube will be transported to the central laboratory for HbA1c and CBC testing uncentrifuged.
4 mL EDTA whole blood tube	The tube will be gently inverted to mix. It is not centrifuged. Blood is aliquoted into 5 mL cryovials and transported to the central laboratory for immediate storage at −80°C for future genetic testing.

CBC, complete blood count; HbA1c, glycated haemoglobin; NaF, sodium fluoride.

### Definitions

Diabetes will be defined according to WHO guidelines as a fasting venous capillary plasma glucose ≥7.0 mmol/L, a 2-hour post-load venous plasma glucose ≥11.1 mmol/L, HbA1c ≥48 mmol/mol (6.5%) or on oral or insulin treatment for diabetes. Pre-diabetes will be defined as a fasting venous or capillary plasma glucose between 5.6 and 6.9 mmol/L, a 2-hour post-load venous plasma glucose between 7.8 and 11.0 mmol/L or HbA1c between 42 and 47 mmol/mol (6.0–6.4%).^13–15^

### Sample size determination

This study is powered to estimate the prevalence of diabetes and pre-diabetes with a high degree of precision. We base our assumptions on results from the 2014 Ugandan nationwide cross-sectional survey suggesting that the prevalence of impaired fasting glucose and diabetes in Uganda is 2% (95% CI: 1.5% to 2.5%) and 1.4% (95% CI: 0.9% to 1.9%), respectively.^16^

Applying a rule of thumb that the margin of error should not exceed 0.25 of the prevalence, and based on the following assumptions: (1) a margin of error of 0.5% (a margin of error of 0.5–1% would be acceptable for low prevalence conditions), (2) diabetes prevalence of 5% (assuming OGTT would detect twice more cases of diabetes thus doubling the prevalence) and an estimated 5% level of significance (z-value=1.96).

The minimum sample size is 7300 for a precision of ±0.5% and a significance level of 5%. Assuming a response rate of 80% and further adjusting for a design effect at 1.2, the required sample size of (7300/0.8)×1.2=10 950. Therefore, we will aim to randomly screen a minimum of 10 950 adults across all study sites.

### Data management

Data management responsibilities for this study will be performed by the MRC/UVRI and LSHTM Uganda Research Unit under the overall supervision of the research study data manager. Study data will be managed electronically using REDCap (Research Electronic Data Capture), a clinical data management system hosted at the MRC/UVRI and LSHTM Uganda Research Unit. REDCap is a secure, web-based application that supports data capture for research studies. This system will be used to create: (1) an intuitive interface for validated data entry, (2) audit trails for tracking data manipulation and export procedures, and (3) automatic data validation checks. Accruing data will be monitored daily, and queries will be generated and shared with the research team by the study data manager weekly. In addition, monthly data reports will be shared to monitor study progress.

### Statistical considerations

Statistical analysis will be performed using STATA V.17 (StataCorp, Texas, USA). All variables will be tabulated to assess distributions and to check for missingness and data sparsity. Using descriptive statistics, categorical variables will be summarised as frequencies and percentages. Continuous variables will be summarised as means and SDs or medians and IQRs. Descriptive analysis will include key sociodemographic outcome variables, for example, education level, socioeconomic status, baseline clinical characteristics (age, sex, weight, BMI and waist circumference) and glycaemic control characteristics (fasting glucose, 2-hour OGTT, HbA1c, fasting C peptide level).

Prevalence of diabetes will be computed as the number of diabetic cases at the end of the study period as a proportion of the total number of participants enrolled into the study. Similarly, prevalence estimates will be used to measure the burden of the different vascular complications within this cohort. We will use Poisson regression analysis to estimate the incidence rate of diabetes-related complications, including retinopathy, nephropathy and neuropathy among individuals with diabetes and pre-diabetes. The reproducibility of the diagnostic tests will be assessed by applying appropriate diagnostic reproducibility statistics, including estimation of the dispersion of the data values on index and repeat testing, interclass correlation and paired t-test (two tailed). Furthermore, we will assess for percentage agreement using Bland-Altman’s methods between the different tests. Before carrying out the analysis, test assumptions will be checked. A two-sided p value of <0.05 will be considered a statistically significant level for all analyses.

### Ethics and dissemination

This study protocol was approved by the UVRI Research Ethics Committee (REC) (number: G.C./127/21/09/858), the LSHTM REC (number: 26638) and the Uganda National Council for Science and Technology (protocol number: HS1791ES). Written informed consent will be obtained from all participants before conducting study procedures. Participants must provide consent for all study procedures to be eligible to participate in the study, including consenting for biospecimens to be stored for future research purposes. Consent to publication will be obtained as part of consent to participation in the study. Research findings will be disseminated in policy briefs, seminars, local and international conferences and publications in peer-reviewed open-access journals. As part of the dissemination plans, findings will also be disseminated to patient care groups and to clinicians in the participating hospitals.

## Discussion

This study aims to determine the prevalence of diabetes and pre-diabetes and establish a cohort of patients with diabetes to understand the course of these different phenotypes, including how people living with diabetes respond to treatment. We expect these data to be of direct relevance to an improved understanding of the pathophysiological basis of diabetes, the performance of diagnostic tests and management of diabetes in SSA, and ultimately to lead to better outcomes and well-being of patients and increased productivity. This protocol has been developed based on standard guidelines for diagnosing and managing individuals with diabetes and sought input from experts in this disease area.

Based on the considerable sample size to be enrolled in this study, we expect to generate a large population-based dataset that will provide a reliable estimate of the burden of the disease within this population. To minimise bias within our study, (1) all the research team members have been well trained on the research study protocol, (2) research assistants have been trained on the administration of the questionnaires, (3) checks have been implemented within the electronic case report form to minimise on the missingness of data related to questionnaire administration, and (4) measures have been put in place to minimise loss to follow-up during the follow-up phase of the study. In addition, unresponsiveness was accounted for during the sample size calculation.

### Study status

This manuscript describes version 1.2 of our protocol. Enrolment in the study began in January 2022. The anticipated end to patient enrolment is November 2023.
